# Integrated datasets on transformational leadership attributes and employee engagement: The moderating role of job satisfaction in the Fast Moving Consumer Goods (FMCG) industry

**DOI:** 10.1016/j.dib.2018.06.032

**Published:** 2018-07-04

**Authors:** Odunayo Salau, Olumuyiwa Oludayo, Hezekiah Falola, Maxwell Olokundun, Stephen Ibidunni, Tolulope Atolagbe

**Affiliations:** Covenant University, Nigeria

**Keywords:** Leadership, Engagement, Management, Traits, Reward, Innovation, Satisfaction

## Abstract

Transformational leadership has gained a great deal of attention since its development through research and evaluations from James MacGregor Burns and Bernard Bass. But central to its development, there are still uncertainties regarding the attributes and interventions of transformational leadership style in developing nations like Nigeria. Hence, this article presented an integrated datasets on transformational leadership attributes, employee satisfaction and engagement using selected Fast Moving Consumer Goods (FMCGs) firms in Nigeria. The study adopted a descriptive research design to establish trends and the quantitative approach was employed using survey questionnaire. A sample size of three hundred and fifty-nine (359) staff and management of sampled firms were selected. Data was analysed with the use of measurement and structural equation modelling and the field data set is made widely accessible to enable critical or a more comprehensive investigation. The findings identified intellectual stimulation (creativity and innovation) and attributed charisma as predictive determinants of transformational leadership attributes for increased satisfaction and engagement of sampled firms. It was recommended that FCMGs firms need to invest necessary resources in developing strategies and interventions to identify differing needs, abilities, and aspirations for staff satisfaction.

## 1 Introduction

Transformational leadership is no longer a new topic in leadership and change management but despite the attention paid to the practice, organisations still fail to understand and explain how leaders can encourage, inspire and motivate employees to innovate and create change that will help grow and shape the future success of the organisations. The theoretical, methodological as well as practical gaps churned out from extant literature are more applicable to the developed and transitional economies. Unfortunately, studies focusing on the contextual and organizational variables in developing economies, such as Nigeria, are still scarce. Hence, this paper adds to knowledge in the area of leadership research as it investigates leadership transformational attributes on employee satisfaction and engagement across hierarchical levels in Nigeria. This research broadens the scope of previous research and provides a more detailed examination of transformational leadership attributes by exploring the five traits –- Idealized influence (II), Inspirational motivation (IM), Intellectual stimulation (IS), Individualized consideration (IC) and Attributed charisma (AC) as presented in Table 1.

**Specification Table**

**Table:** 

**Subject area**	Business Management
**More Specific Subject Area**	Strategic Leadership
**Type of Data**	Primary data
**How Data was Acquired**	Through questionnaire
**Data format**	Raw, analyzed, Inferential statistical data
**Experimental Factors**	The researcher-made questionnaire which contained data on transformational leadership attributes, employee satisfaction and engagement using selected FMCGs firms
**Experimental features**	This paper contributes to the shift in focus by exploring the contextual impact of employee satisfaction as a moderating variable on transformational leadership and employee engagement across hierarchical levels of sampled firms
**Data Source Location**	Lagos, Nigeria
**Data Accessibility**	Data is included in this article

**Value of the data**

• The data can be used to explain how managers and their followers can properly raise one another to higher levels of morality and motivation.
• The data can be used to enlighten managers on the importance of motivating subordinates to produce superior performance without any form of coercive authority/ power
• The data provides ample knowledge on how different transformational leadership attributes would in turn lead to job satisfaction and engagement
• The data described in this article is made widely accessible to facilitate critical or extended analysis.

## 2 Data

The study is quantitative in nature and data were retrieved from staff and management of sampled firms. The questionnaire was divided into two [2] sections. An extensive list of items in the questionnaire was developed to understand the nature and the type of transformational leadership style practiced by the sampled firms [1], [4], [5], [6], [11]. The first section (A) captures the demographic characteristics of the population, section B focused on questions to measure each specific objectives of the study. The questionnaire was structured, with both close and open-ended questions in order to get free expression of ideas among the respondents.

Of the 400 copies of questionnaire that were sent to the selected FCMG firms, 359 of these staff participated in the survey representing a response rate of 89%. To achieve this response rate, several follow-up attempts were made to increase participation in the research. These included personal phone calls, as well as sending follow-up emails to the leaders asking for participation. The data generated from questionnaire were checked for completeness and was later converted and processed into useful information. The quantitative survey posed closed questions with the answer options provided. First, the responses were given numerical codes using a code book. Second, codes were translated into a computer readable material called the “coding sheet”. From the coding sheet; the data were analysed with the aid of the Statistical Package for Social Sciences (SPSS), version 22.

To avoid confusion or problem of interpretation by the respondents, the questionnaire were prepared in a simple and clear language to avoid ambiguity (see Appendix). Table 1 describes the Four I׳s and One A— leaders who display transformational leadership behaviours and traits as indicated by MacGregor Burns & Bernard Bass.

**Table 1:** A display of transformational leadership behaviours and traits.

Idealized Influence (II)	• Instil pride in followers (charismatic) • Goes beyond their self-interest for the greater good of the organization • Displays a sense of power and confidence • Talk about their most important values and beliefs • Emphasize collective mission
Inspirational Motivation (IM)	• Talk optimistically about future • Articulate a compelling vision for the future • Talk about what needs to be accomplished; express confidence that goals will be achieved • Creates exciting image of what is essential to consider • Encourages team-spirit, general enthusiasm
Intellectual Stimulation (IS)	• Seeks differing perspectives • Gets others to look at problems from differing angles • Encourage non-traditional thinking • Suggest new ways of looking at completing assignments • Re-examine critical assumptions
Individualized Consideration (IC)	• Spend time coaching and teaching followers • Promote self-development • Treat team members as individuals • Identify differing needs, abilities, and aspirations for team members • Listen to others’ concerns • Help develop others’ strengths
Attributed Charisma (AC)	• Attract followers and inspires people to action • Initiate and maintain a significant level of change in the organization • Behave in a mature and responsible manner on all occasions. • Place a lot of value and have the ability to truly listen to employees. • Motivate subordinates to produce superior performance without the use of formal authority or power • Inspire and enthuse their subordinates through their articulation of an organisational vision

The decision to elicit information from the employees and the management group was based on the fact that while employees were often in the best position to describe their leaders’ traits and behaviours; it is also crucial to investigate these practices from the perceptions of the managers. This shows that the samples were diverse and it can be concluded that non-response bias will not significantly affect the generalizability of the study findings. The analysis was also carried out to identify the attributes/benefits influencing transformational leadership attributes in the sampled firms.

During the data collection stage, demographic variables age, experience, and education were all coded, or scaled, so that the numbers shown do not reflect actual numbers. The scales used to code each of these variables is shown in Table 2.

**Table 2:** Demographic variable measurements.

Value	Education	Experience	Age	Marital Status	Gender
1	No formal education	< 1 year	< 20year	Single	Male
2	Primary education	1–5 years	21–30 years	Married	female
3	Secondary education	6–10 years	31–40 years	Divorced	
4	BSc./HND	> 10 years	> 40 years	Separated	
5	MSc./MEd.				
6	PhD.				

**Fig. 1:**
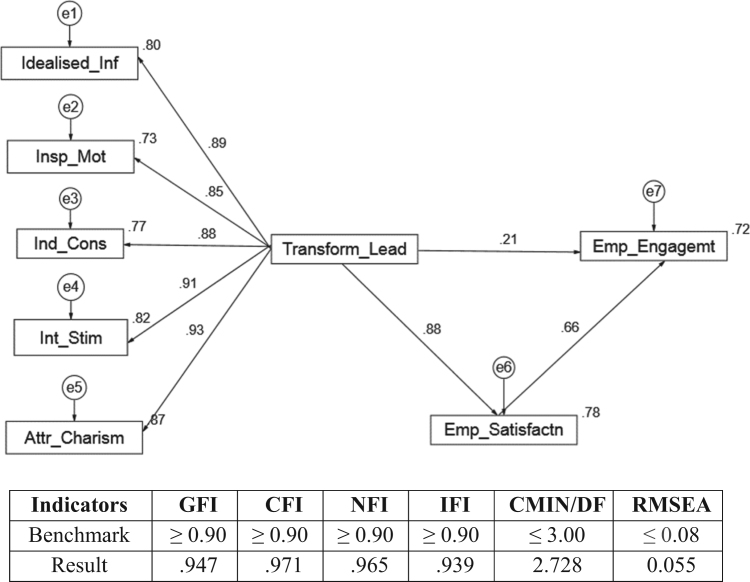
Regression weight of the variables.

Fig. 1 illustrates how the moderating variable in this study may influence the engagement of employees in the selected Fast Moving Consumer Goods. In other words, the employee satisfaction was analyzed to evaluate if any of them changed the relationship between transformational leadership attributes and the engagement of employees over time as presented in Fig. 1.

The data met the conditions of basic assumption in SEM analysis. The result indicates that there are varying explanations for the dependent variables. As recommended by MacGregor Burns & Bernard Bass, five measures and MLQ-5× subscales were used to calculate the score for each individual. These measures were identified as Idealized Influence (II), Inspirational Motivation (IM); Intellectual Stimulation (IS); Individualized Consideration (IC); and Attributed Charisma (AC). Factor loading was conducted to ensure the validity and reliability of the research items as presented in Table 3.

**Table 3:** Factor loadings of the research items.

**Measurement**	**Loading**	**Indicator Reliability**	**Error Variance**	**Compose Reliability**	**Ave. Variance Estimated**
	**> 0.7**		**< 0.5**	**> 0.8**	**> 0.5**
**Transformational Leadership Style**					

*Idealized Influence (Purpose Driven)*					
II1	0.817	0.6675	0.3325	0.9049	0.7043
II2	0.823	0.6773	0.3227		
II3	0.898	0.8064	0.1936		
II4	0.816	0.6659	0.3341		

*Inspirational Motivation (Encouragement and Rewards)*					
IM1	0.861	0.7413	0.2587	0.8722	0.6318
IM2	0.831	0.6906	0.3094		
IM3	0.777	0.6037	0.3963		
IM4	0.701	0.4914	0.5086		

*Individualized Consideration (People Driven)*					
IC1	0.853	0.7276	0.2724	0.8608	0.6105
IC2	0.646	0.4173	0.5827		
IC3	0.871	0.7569	0.2431		
IC4	0.735	0.5402	0.4598		

*Intellectual Stimulation (Creativity And Innovation)*					
IS1	0.908	0.8245	0.1755	0.9412	0.8002
IS2	0.916	0.8391	0.1609		
IS3	0.841	0.7073	0.2927		
IS4	0.911	0.8299	0.1701		

*Attributed Charisma (AC)*					
AC1	0.759	0.5761	0.4239	0.9086	0.7143
AC2	0.803	0.6448	0.3552		
AC3	0.902	0.8136	0.1864		
AC4	0.907	0.8226	0.1774		

## 3 Experimental design, materials and methods

Based on the definite population sample determination formula stated in the sampling techniques section, a total of 359 copies of questionnaire were planned for distribution. A total of 359 out of 400 staff and leaders of the sampled FCMGs firms participated in the current study, accounting for 89%. The decision to explore the attitudes of both the employees and the management group on transformational leadership attributes, employee satisfaction and engagement was based on the fact that while employees were often in the best position to describe the real attributes of their managers/leaders/supervisors; it is also crucial to investigate these practices from the perceptions of the managers.

The data generated through quantitative approach involved the use of structured questionnaire. Overall, the survey instrument has a total of twenty-one 21 questions, divided into two broad sections; Section A (1–6) contains basic socio-demographic questions of age, sex, age, ethnicity, religion etc. while Section B (7–21) covers questions relating to transformational leadership attributes and these items in the questionnaire were adopted based on the works of [3], [7], [8], [9], [10], and [12].

For the purpose of efficiency and thoroughness two field assistants were recruited and trained. The training helped the research assistants to familiarize themselves with the research instrument and objectives of the study before going to the field. This also helped the researcher to make sure that they understand all the questions in the questionnaire, so as to minimize error and enhance the quality of data collected. The training enabled them acquire skills and helped them to effectively interact with the respondents.

Each questionnaire was checked on the spot to ensure that they were properly filled. Transformational leadership practices were measured using items adapted from previous studies. The study used a between-groups design with 7 dependent variables. These variables were - Attributed charisma (AC), Idealized influence (II), Inspirational motivation (IM), Intellectual stimulation (IS), Individualized consideration (IC), Transformational leadership (TFL) (a composite of the preceding five variables), Employee satisfaction (ES) and engagement (EE). All of these variables reflected scales in the MLQ. It is vital that the MLQ yields an accurate and unbiased assessment of leaders on the various leadership dimensions.

Questionnaire for the study were sorted and those that were not properly filled were removed. To minimize errors, data from questionnaire were coded so as to pave way for editing of data before the use of SPSS-Statistical package for Social Sciences-software. The demographic data presented information based on gender, age, education and experience as well as questions related to transformational leadership attributes, employee satisfaction and engagement. There was a meaningful relationship among transformational leadership attributes, satisfaction and engagement of staff in the selected firms.

The type of scales of measurement (nominal, ordinal and scale) and the questionnaire format (close and open ended questions) necessitates the statistical analysis at univariate, bivariate and multivariate levels and with the aid of the Statistical Package for the Social Sciences (SPSS v22.0). Univariate analysis was carried out through descriptive statistics of frequency and percentages while bivariate and multivariate analysis were achieved through the measurement model and structural model.

This study revealed that transformational leadership attributes (TLA) has significant and positive impact on job satisfaction and engagement. By fostering an atmosphere of reassurance, managers and supervisors need to tie the vision to a strategy for its achievement and develop a challenging and attractive vision, together with the employees. Hence, this present study has extensive implications for both the managers, employees, government, educators and researchers in this regard. To this end, the data presented in this article is imperative for more comprehensive investigation. The researchers ensured that respondents were well informed about the background and the purpose of this research and they were kept abreast with the participation process. All persons and organizations relevant to study objectives and having relevant information and data were consulted and necessary clearances were formally sought. Respondents were offered the opportunity to stay anonymous and their responses were treated confidentially.

## Footnotes

Appendix A Supplementary data associated with this article can be found in the online version at https://doi.org/10.1016/j.dib.2018.06.032.

## References

[1] Alban-Metcalfe R.J., Alimo-Metcalfe B. (2000). The transformational leadership questionnaire (TLQ-LGV): a convergent and discriminant validation study. Leadersh. Organ. Dev. J..

[2] Bakker A.B., Schaufeli W.B., Leiter M.P., Taris T.W. (2008). Work engagement: an emerging concept in occupational health psychology. Work Stress.

[3] Bass B.M. (1995). Theory of transformational leadership redux. Leadersh. Q..

[4] Bass B.M., Riggio R. (2006). Transformational Leadership.

[5] Bolkan S., Goodboy A.K. (2011). Behavioral indicators of transformational leadership in the college classroom. Qual. Res. Rep. Commun..

[6] Falola H.O., Salau O.P., Olokundun A.M., Oyafunke-Omoniyi C.O., Ibidunni A.S., Oludayo O.A. (2018). Employees׳ intrapreneurial engagement initiatives and its influence on organisational survival. Bus.: Theory Pract..

[7] Ibidunni O.S., Osibanjo A.O., Adeniji A.A., Salau O.P., Falola H.O. (2016). Talent retention and organizational performance: a competitive positioning in Nigerian Banking Sector. Period. Polytech. Social. Manag. Sci..

[8] McBain R. (2007). The practice of engagement: research into current employee engagement practice. Strat. HR Rev..

[9] Northouse P.G. (2016). Leadership: Theory and Practice.

[10] Rafferty A.E., Griffin M.A. (2004). Dimensions of transformational leadership: conceptual and empirical extensions. Leadersh. Q..

[11] Saks A.M. (2006). Antecedents and consequences of employee engagement. J. Manag. Psychol..

[12] Schriesheim C.A., Wu J.B., Scandura T.A. (2009). A Meso measure? Examination of the levels of analysis of the Multifactor Leadership Questionnaire (MLQ). Leadersh. Q..

