# Supplementary material for: Integrated datasets on transformational leadership attributes and employee engagement: The moderating role of job satisfaction in the Fast Moving Consumer Goods (FMCG) industry
Source: Data Brief. 2018 Jul 4;19:2329–35. doi: 10.1016/j.dib.2018.06.032 (PMC6141153; doi:10.1016/j.dib.2018.06.032)
Supplement: Supplementary file 1 — Supplementary material [file mmc1.docx]

**DECLARATION OF INTEREST FORM**

[**Integrated Datasets on Transformational Leadership Attributes and Employee Engagement: The Moderating role of Job Satisfaction in**](https://www.sciencedirect.com/science/article/pii/S2352340917304353) **the Fast Moving Consumer Goods (FMCG) Industry**

Odunayo **SALAU**; Covenant University

[odunayo.salau@covenantuniversity.edu.ng](mailto:odunayo.salau@covenantuniversity.edu.ng)

Olumuyiwa **OLUDAYO**; Covenant University

olumuyiwa.oludayo[@covenantuniversity.edu.ng](mailto:ebe.igbinoba@covenantuniversity.edu.ng)

Hezekiah **FALOLA**; Covenant University

[hezekiah.falola@covenantuniversity.edu.ng](mailto:hezekiah.falola@covenantuniversity.edu.ng)

Maxwell **OLOKUNDUN**; Covenant University

maxwell.olokundun[@covenantuniversity.edu.ng](mailto:hezekiah.falola@covenantuniversity.edu.ng)

Stephen **IBIDUNNI**; Covenant University

stephen.ibidunni[@covenantuniversity.edu.ng](mailto:hezekiah.falola@covenantuniversity.edu.ng)

Tolulope **ATOLAGBE**; Covenant University

tolulope.atolagbe[@covenantuniversity.edu.ng](mailto:ebe.igbinoba@covenantuniversity.edu.ng)

We, the Authors of paper entitled above certify that we have seen and approved the final version of the manuscript being submitted. This is an original work and has not received prior publication and is not under consideration for publication elsewhere. It is also important to state that there is no financial/personal interest or belief that could affect our objectivity and to prevent ambiguity, we humbly want to state explicitly that there is no conflicts of interest as regards the review and publication of this paper.

Thank you.

SALAU Odunayo Paul

*Signed*
