# Supplementary material for: Integrated datasets on transformational leadership attributes and employee engagement: The moderating role of job satisfaction in the Fast Moving Consumer Goods (FMCG) industry
Source: Data Brief. 2018 Jul 4;19:2329–35. doi: 10.1016/j.dib.2018.06.032 (PMC6141153; doi:10.1016/j.dib.2018.06.032)
Supplement: Supplementary file 3 — Supplementary material [file mmc3.docx]

**RESEARCH QUESTIONNAIRE**

Department of Business Management,

Covenant University P.M.B.1023, Ota, Ogun State.

Dear Respondent,

We are researchers from the above named institution conducting a research on “**Transformational Leadership Attributes and Employee Engagement: The Moderating role of Job Satisfaction**. Kindly respond appropriately to each section of the questionnaire. Feel free to give your sincere opinion and feelings as demanded by each question. Your response will be kept in absolute confidence. It is purely for academic purpose.

Thank you.

**SECTION A**: **SOCIO DEMOGRAPHIC CHARACTERISTICS OF RESPONDENTS**

Instruction: Kindly tick ( ) and fill where applicable.

1. Name of Firm/Organisation: _______________________________________________
2. Gender: Male ( ) Female ( )
3. Marital status: Single ( ) Married ( ) Divorced ( ) Divorced ( ) Separated ( )
4. Age: < 20year ( ) 21-30 years ( ) 31-40 years ( ) > 40 years ( )
5. Years of Service/Experience: < 1 year ( ) 1-5 years ( ), 6-10 years ( ) > 10 years ( )
6. Highest Educational Qualification: No formal Education ( ) Primary Education ( ) Secondary education ( ) Bachelor’s Degree ( ) Master’s Degree ( ) Ph.D ( )

**SECTION A: Transformational Leadership Attributes**

**Instruction**: Please indicate on a scale of 1 to 5; If you Strongly Agree (SA), for instance, tick 1, or if you Strongly Disagree (SD), please tick 5. We are interested in the number that best shows your views.

|  | **Items** | **SA** | **A** | **U** | **D** | **SD** |
| --- | --- | --- | --- | --- | --- | --- |
| **Leaders in my organisation:** | | | | | | |
| Q1 | go out of the way to make others feel good to be around me | 1 | 2 | 3 | 4 | 5 |
| Q2 | help others with their self-development. | 1 | 2 | 3 | 4 | 5 |
| Q3 | help others to understand my visions through the use of tools, such as images, stories, and models | 1 | 2 | 3 | 4 | 5 |
| Q4 | ensure others get recognition and/or rewards when they achieve difficult or complex goals | 1 | 2 | 3 | 4 | 5 |
| Q5 | let others work in the manner that they want | 1 | 2 | 3 | 4 | 5 |
| Q6 | have an ever-expanding network of people who trust and rely upon me | 1 | 2 | 3 | 4 | 5 |
| Q7 | provide challenges for my team members to help them grow | 1 | 2 | 3 | 4 | 5 |
| Q8 | use simple words, images, and symbols to convey to others what we should or could be doing |  |  |  |  |  |
| Q9 | manage others by setting standards that we all agree with |  |  |  |  |  |
| Q10 | rarely give direction or guidance to others if I sense they can achieve their goal |  |  |  |  |  |
| Q11 | consistently provide coaching and feedback so that my team members know how they are doing |  |  |  |  |  |
| Q12 | People listen to my ideas and concerns not out of fear, but because of my skills, knowledge, and personality |  |  |  |  |  |
| Q13 | provide an empathic shoulder when others need help |  |  |  |  |  |
| Q14 | help others with new ways of looking at new and complex ideas or concepts. |  |  |  |  |  |
| Q15 | ensure poor performance is corrected |  |  |  |  |  |
| Q16 | monitor all projects that I am in charge of to ensure the team meets it goal. |  |  |  |  |  |
| Q17 | promotes others to share ideas |  |  |  |  |  |
| Q18 | challenges others to try new ideas |  |  |  |  |  |
| Q19 | gives ethical consideration to actions |  |  |  |  |  |
| Q20 | promotes attainment of the vision |  |  |  |  |  |

**Specific Attributes of Transformational Leadership**

This section focuses on the specific attributes of transformational leadership in your organisation. To the best of your knowledge. Please circle the number or point that represents your view.

|  | ITEMS | **Lowest 100%**  **1-20%** | **Lower 100%** **21-40%** | **Middle 100%** **41-60%** | **Close 100%** **61-80%** | **Top 100%**  **81-100%** |
| --- | --- | --- | --- | --- | --- | --- |
| 1 | Ability to develop a vision | 1 | 2 | 3 | 4 | 5 |
| 2 | Ability to develop others | 1 | 2 | 3 | 4 | 5 |
| 3 | Ability to support others | 1 | 2 | 3 | 4 | 5 |
| 4 | Ability to take sensible risk | 1 | 2 | 3 | 4 | 5 |
| 5 | Giving ethical consideration | 1 | 2 | 3 | 4 | 5 |
| 6 | Ability to prepares for change | 1 | 2 | 3 | 4 | 5 |
| 7 | Promoting idealization of leader |  |  |  |  |  |

**SECTION B: Employee Satisfaction** (ES)

|  | **Items** | **SA** | **A** | **U** | **D** | **SD** |
| --- | --- | --- | --- | --- | --- | --- |
| Q1 | I feel encouraged to come up with new and better ways of doing things. | 1 | 2 | 3 | 4 | 5 |
| Q2 | My work gives me a feeling of personal accomplishment. | 1 | 2 | 3 | 4 | 5 |
| Q3 | The firm does an excellent job of keeping employees informed about matters affecting us. | 1 | 2 | 3 | 4 | 5 |

**SECTION C: Employee Engagement** (EE)

|  | **Items** | **SA** | **A** | **U** | **D** | **SD** |
| --- | --- | --- | --- | --- | --- | --- |
| Q1 | My job makes good use of my skills and abilities. | 1 | 2 | 3 | 4 | 5 |
| Q2 | My firm gives a clear understanding of my career or promotion path | 1 | 2 | 3 | 4 | 5 |
| Q3 | My firm encourages the need to [balance work and our personal life](https://www.tinypulse.com/blog/sk-achieve-work-life-balance) in order to be productive | 1 | 2 | 3 | 4 | 5 |
